# Local Uses of Tannin

**Published:** 1872-10

**Authors:** 


Local Uses of Tannin.—Dr. G. P. Hachenberg (*New-York Medical Record*, August 15, 1872), reports several cases of the uses of this remedy in prolapsus uteri, where other means had failed to afford relief. His method is as follows: A glass speculum is introduced into the vagina so as to push the uterus into its place. Through the speculum a metallic tube or syringe, with the end containing about thirty grains of tannin, is passed. With a piston, the tannin is now pushed against the uterus, the syringe withdrawn, and the packing neatly and effectually completed, with a dry probang around the mouth and neck of the womb. After the packing is completed, the probang is placed against the tannin, in order to hold it, and the speculum is partially withdrawn. The packing is now fully secured and the instrument removed.

The application of tannin holds the uterus firmly and securely in place, not by dilatation of the walls of the vagina, but by corrugating and contracting its parts. At first, the applications may be made weekly; finally, but once or twice a month. It not only overcomes the hypertrophy and elongation of the cervix, but even, the writer thinks, induces a slight atrophy of the parts. As a remedy for leucorrhœa, where the seat of the inflammation is at the mouth of the womb, or within the vagina, it actually gives speedy relief.

Dr. G. P. Hachenberg also reports a case of chronic ulceration of the rectum, which was cured after a few weekly packings of tannin.

He has found, moreover, that in affections of the throat, direct applications of tannin to the diseased parts give satisfactory results. In a case of extraordinary hypertrophy of the tonsils, preparatory to the operation of extirpation, tannin, mixed with tincture of iodine to the consistency of syrup, was applied with the effect ot so diminishing the hypertrophy that a surgical operation will in all probability not be necessary.

No remedy has given such satisfactory results in certain forms of chronic ophthalmia and opacity of the cornea as tannin. Once a week place under the eyelids pure, well-triturated tannin. The application is not very painful, and the tears soon dissolve the tannin. An aged lady, who had chronic ophthalmia, was relieved by one application; another, who was blind from opacity of the cornea and chronic ophthalmia, recovered her sight mainly from the local use of powdered tannin—*Boston Medical and Surgical Journal.*

